# The mitochondrial ATP synthase as an ATP consumer—a surprising therapeutic target

**DOI:** 10.15252/embj.2023114141

**Published:** 2023-04-06

**Authors:** Gabriel E Valdebenito, Anitta R Chacko, Michael R Duchen

**Affiliations:** ^1^ UCL Consortium for Mitochondrial Research and Department of Cell and Developmental Biology University College London London UK

**Keywords:** Metabolism, Molecular Biology of Disease

## Abstract

The mitochondrial F_1_F_o_‐ATP synthase uses a rotary mechanism to synthesise ATP. This mechanism can, however, also operate in reverse, pumping protons at the expense of ATP, with significant potential implications for mitochondrial and age‐related diseases. In a recent study, Acin‐Perez *et al* (2023) use an elegant assay to screen compounds for the capacity to selectively inhibit ATP hydrolysis without affecting ATP synthesis. They show that (+)‐epicatechin is one such compound and has significant benefits for cell and tissue function in disease models. These findings signpost a novel therapeutic approach for mitochondrial disease.

## Graphical abstract

Recent work shows that preventing the F_1_F_o_‐ATP synthase from operating in reverse could be a new way of treating mitochondrial diseases.

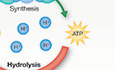

The mitochondrial F_1_F_o_‐ATP synthase (also called complex V) is a remarkable enzyme and is fundamental for eukaryotic life. The enzyme sits in the mitochondrial inner membrane and acts as a turbine, harnessing the energy generated by respiration in the form of ATP. The accumulation of ATP in the mitochondrial matrix is avoided through the equilibration of ATP with the cytosol through the Adenine Nucleotide Translocator (ANT), which brings ADP into the matrix and ensures ATP supply in the cytosol where it is used to power everything from ion homeostasis to protein synthesis and from secretion to motility (Duchen, 2004).

This molecular motor will run in either direction, depending on the balance between the free energies of the (ATP/ADP+P_i_) ratio and the proton motive force generated by the respiratory chain. The prevailing view would state that normally respiring mitochondria generate a mitochondrial membrane potential of around 180 mV. These conditions should favour the activity of complex V as a synthase, while the possibility of reversal as key variable change has been described by a mathematical model (Metelkin *et al*, 2009).

The capacity of the molecular motor to run in “reverse” as an ATP‐consuming proton pump is most readily seen after inhibition of the respiratory chain or exposure to uncouplers. In cells with a strong capacity to upregulate glycolysis such as astrocytes (Almeida *et al*, 2004), exposure to respiratory chain inhibitors such as cyanide or rotenone induces only small and slow changes in membrane potential, as the mitochondrial membrane potential is maintained by the reversal of the F_1_F_o_‐ATPase: ATP generated by glycolysis is consumed, and protons are pumped across the mitochondrial inner membrane at a rate that is sufficient to maintain the potential. Repeat of the experiment following inhibition of the ATPase with oligomycin induces a rapid and complete mitochondrial depolarisation (Jacobson & Duchen, 2002), illustrating the power of the ATPase as a proton pump. The impact of the ATPase as an ATP consumer is most dramatically illustrated by rat cardiomyocytes, a cell type with a very high density of mitochondria. Following mitochondrial depolarisation, at reperfusion injury or in response to uncoupler, ATP is consumed and completely depleted within minutes and the cell will go into a rigour contracture (Bowers *et al*, 1992). However, in the presence of oligomycin, the cell will just sit with depolarised mitochondria for an hour without any significant loss of ATP (Allue *et al*, 1996; Leyssens *et al*, 1996).

Cells have evolved a mechanism to defend themselves against ATP depletion mediated by the mitochondrial ATPase. This mechanism involves the protein ATPIF1 or the “endogenous inhibitor protein.” Discovered in the 1960s (Pullman & Monroy, 1963), ATPIF1 binds to the ATPase when the matrix pH falls, as happens if respiration is compromised. Acting like a stick in the spokes of a bicycle wheel, it prevents the rotation of the molecular motor and limits the rate of ATP hydrolysis (Bason *et al*, 2011). ATPIF1 appears not to be expressed strongly in small rodents—hence our specific earlier reference to rat cardiomyocytes—but it is strongly expressed in the heart of larger mammals (Rouslin & Broge, 1996). We see the power of ATPIF1 in cardiomyocytes from the human atrial appendage, which shows no rapid progression to rigour contracture in response to uncoupler (Shanmuganathan *et al*, 2005).

Recent work of the Shirihai group demands some refinement of these views and points to a more subtle model. They first showed that super‐resolution imaging of the distribution of the potential‐dependent probe tetramethylrhodamine methyl ester (TMRM) revealed that membrane potentials may vary between different cristae even within a single mitochondria (Wolf *et al*, 2019). This discovery led to the idea that, even at the level of a single mitochondrion, the efficiency of ATP production may vary between cristae. Some cristae may even be running the ATPase “in reverse,” simultaneously consuming ATP that is being generated by their neighbours. This would imply that the net rate of ATP production by a mitochondrion or a cellular mitochondrial population could represent a balance between populations of ATP synthases running either in “forward” or in “reverse” modes. The net efficiency of such a process in healthy cells would then depend on the expression of ATPIF1—which seems to vary between cell types (Campanella *et al*, 2009).

In disease models in which oxidative respiration, and hence mitochondrial membrane potential, is compromised, net ATPase activity can reverse. As explained above, this maintains mitochondrial membrane potential at the expense of ATP consumption (Fig 1). This seems to happen in many disease models and is again revealed experimentally as, under these conditions, exposure to oligomycin leads to the progressive loss of membrane potential (oligomycin normally causes a small increase in potential). Now, Acin‐Perez *et al* (2023) propose that this mitochondrial ATP consumption may be a maladaptive response, driving energy depletion and contributing to the disease phenotype and progression. They therefore sought to target the reverse activity of the molecular motor, developing a screening protocol to identify a compound, (+)‐epicatechin (EPI), which appears selectively to inhibit ATPase activity without affecting ATP synthase activity. In their paper published in this issue of The EMBO Journal, Acin‐Perez *et al* (2023) show that exposure of cells to EPI increased net ATP production in a number of cell models. Furthermore, they show that treatment of *mdx* mice, which express a form of muscular dystrophy, with EPI resulted in improved exercise performance and increased life span. These data are interesting on many levels. In diseases that cause impaired respiration, it has been unclear which is more important in terms of pathophysiology: to preserve ATP levels (especially in cells with a limited capacity to upregulate glycolysis) or to maintain mitochondrial membrane potential (at the expense of cellular ATP). Which strategy might have more severe long‐term consequences? The appearance of ATPIF1 argues for the former, and this is supported by the work reported by Acin‐Perez *et al* (2023), as the long‐term treatment of cells, or even animals, with a compound that limits mitochondrial ATP consumption seems to mitigate dysfunction associated with disease. These findings also require us to reconsider the processes that dictate net ATP production by cells, and to take into account the possibility of ATP consumption by the ATPase itself, even in healthy cells. We know almost nothing about how ATPIF1 expression is regulated, but these authors also show that levels change in some of the disease models they have explored, suggesting that this is another variable that can be modulated and that should be considered in attempts to understand mitochondrial disease and cellular energy homeostasis.

**Figure 1:**
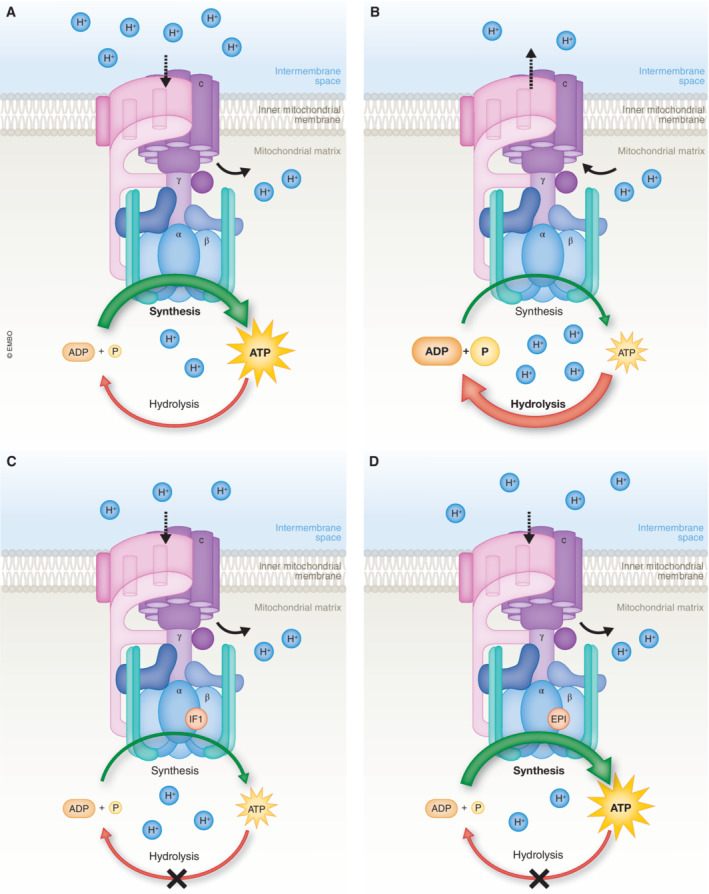
Schematic illustration of the ATP synthase and its regulation (A) Canonical mechanism of the forward mode of the ATP synthase, which involves the conversion of ADP and Pi into ATP. (B) Reversal of the ATP synthase leads to the breakdown of ATP into ADP+P_i_. (C) Endogenous protein ATPIF1 acts as a natural inhibitor of the ATP synthase. (D) The mimetic compound (+)‐Epicatechin binds to the ATPIF1‐binding groove of CV, leading to the subsequent inhibition of ATP hydrolysis, without affecting ATP synthesis.

There are many emerging aspects of mitochondrial research that show us how much we still have to learn in terms of cell signalling and metabolite processing and in understanding mitochondrial disease, and these data highlight that, even in terms of fundamental cellular bioenergetic homeostasis, there remain intricacies and subtleties that we have yet to explore.
